# Supplementary material for: First Report on Microcystin-LR Occurrence in Water Reservoirs of Eastern Cuba, and Environmental Trigger Factors
Source: Toxins (Basel). 2022 Mar 15;14(3):209. doi: 10.3390/toxins14030209 (PMC8952431; doi:10.3390/toxins14030209)
Supplement: Supplementary file 1 [file toxins-14-00209-s001.zip › toxins-1617086-supplementary.pdf]

Supplementary Materials: First Report on Microcystin-LR Occurrence in Water Reservoirs of Eastern Cuba, and Environmental Trigger Factors

José Carlos Rodriguez Tito, Liliana Maria Gomez Luna, Wim Noppe Noppe and Inaudis Alvarez Hubert

Table S1 Database of the 24 studied reservoirs at the eastern Cuba.

Reservoir	Volume	Depth	Catchment area	Inhabitants per km²	Localization		Uses				
	(x 10^6 m^3)	(m)	(km^2)	(Inhabitants)	Longitude	Latitude	Forest (%)	Agric (%)	Ind (%)	Urbanized (%)	Deforest (%)
Céspedes	243,00	52,0	441,91	16345	20.275245	-76.234201	50	35	0	5	10
Gilbert	59,67	43,0	144,10	3002	20.129631	-76.076248	70	20	0	0	10
Gota Blanca	83,60	45,4	319,99	4753	20.164505	-76.031349	70	20	0	0	10
Hatillo	5,84	22,3	108,00	820	20.185080	-70.967581	55	40	0	0	5
Charco Mono	4,50	28,0	72,21	702	20.121330	-75.966522	80	10	0	0	10
Chalóns	3,20	9,0	12,55	4306	20.071577	-75.813723	40	30	0	0	30
Parada	34,20	33,5	55,37	18277	20.034779	-75.896751	50	10	20	10	10
Mícara	4,41	30,6	17,21	501	20.456192	-75.518675	60	20	0	0	20
Joturo	2,38	19,0	20,53	2238	20.183933	-75.574199	30	50	0	0	20
Baraguá	250,00	43,0	1749,61	166498	20.398293	-75.977562	20	60	0	10	10
Camazán	6,70	17,0	54,56	2259	20.768137	-76.112311	20	50	0	10	20
Bio	67,50	25,2	251,95	7256	20.496219	-76.101932	5	90	0	0	5
Cauto el Paso	330,00	30,0	8969,20	1112010	20.584679	-76.718730	40	40	5	5	10
Guisa	66,50	56,0	95,07	21914	20.244891	-76.568438	55	25	0	5	15
Bueycito	159,00	42,0	130,58	12004	20.211159	-76.764833	50	20	0	5	25
Paso Malo	95,60	47,0	229,56	230	20.128539	-76.959294	80	0	0	5	15
Cautillo	84,40	42,0	176,66	10519	20.305233	-76.509774	60	30	0	5	5
La Yaya	160,00	47,2	565,30	35727	20.124841	-75.360015	20	70	0	0	10
Jaibo	120,00	48,5	215,86	2473	20.201719	-75.278045	60	30	0	0	10
Clotilde	6,10	27,0	55,48	396	20.198874	-75.153287	60	20	0	0	20
Los Plátanos	3,80	11,0	22,58	135	20.232075	-75.231228	40	40	0	0	20
Moa	141,00	75,5	86,81	2000	20.557866	-74.981418	40	10	30	10	10
Nipe	112,20	35,0	397,50	10937	20.666997	-75.846526	20	55	0	5	20
Gibara	65,50	46,3	85,97	368	20.901041	-76.113282	30	60	0	0	10

Table S2 List of cyanobacteria species and potentially toxigenic species in the studied reservoirs in eastern Cuba.

Cyanophyceae specie	1	2	3	4	5	6	7	8	9	10	11	12	13	14	15	16	17	18	19	20	21	22	23	24
Aphanizomenon flos-aquae (T)										x	x	x						x		x		x	x	x
Aphanizomenon gracile (T)	x									x	x										x	x		
Aphanocapsa sp. (T)	x	x	x		x	x		x	x				x			x		x	x		x	x	x	x
Aphanothece minutissima	x							x						x	x		x				x	x		x
Aphanotece sp.	x	x		x		x			x								x		x	x				
Aphanothece clathrata	x							x			x	x					x					x		x
Chroococcus minutus									x															
Chroococcus sp.				x					x					x	x		x					x		x
Coelosphaerium sp.								x																
Coelosphaerium kuetzingianum					x																			

Cylindrospermopsis curvispora (T)				x						x*	x											x		x
Cylindrospermopsis raciborskii (T)				x*						x	x	x												
Eucapsis sp.		x			x	x			x	x	x	x												
Geitlerinema splendidum	x	x		x		x				x*	x			x										
Glaucospira sp.										x	x	x			x		x					x		
Gloeocystis vesiculosa						x	x															x		
Gomphosphaeria sp. (T)			x	x	x								x											
Leptolyngbya sp. (T)										x	x	x												
Limnothrix sp. (T)										x	x	x		x										
Merismopedia glauca					x			x	x	x	x	x								x				
Merismopedia punctata					x			x	x	x	x	x				x	x		x	x	x		x	x
Microcystis aeruginosa (T)						x	x*	x*						x			x	x*	x					
Microcystis comperei (T)	x					x			x					x										
Microcystis flos-aquae (T)				x*				x*									x	x				x	x	x
Microcystis panniformis (T)	x			x				x*		x	x													x
Microcystis viridis (T)		x			x		x		x	x			x	x	x		x							
Microcystis wesenbergii (T)		x				x		x*					x		x		x							
Oscillatoria sp. (T)	x	x	x							x*		x*	x				x							
Planktolyngbya limnetica (T)										x		x*					x		x	x				
Planktolyngbya sp. (T)	x	x		x																	x	x		
Planktothrix sp.		x			x	x		x	x			x	x	x	x				x		x		x	x
Pseudanabaena sp. (T)										x	x	x	x		x		x							
Romeria simplex		x			x	x				x	x	x			x	x					x	x	x	x
Spirulina subsalsa										x														
Stanieria cyanosphaera										x		x												
Synechococcus sp. (T)		x				x								x*	x		x							
Synechocystis aquatilis (T)		x			x	x*	x	x		x	x	x	x		x		x	x		x	x	x		
Woronichinia sp. (T)					x																			
Total species	10	12	3	9	11	12	4	12	10	20	16	16	8	9	10	3	15	5	6	6	8	13	6	11

Legend:1-Céspedes; 2-Gilbert; 3-Gota Blanca; 4-Hatillo; 5-Charco Mono; 6-Chalóns; 7-Parada; 8-Mícara; 9-Joturo; 10- Baraguá; 11-Camazán; 12-Bio; 13-Cauto el Paso; 14-Guisa; 15-Bueycito; 16-Paso Malo; 17-Cautillo;18-La Yaya; 19-Jaibo; 20-Clotilde; 21-Los Plátanos; 22-Moa; 23-Nipe; 24-Gibara;.*= Bloom forming species determined in the reservoirs according to the present research;. (T) = toxic species according previous reports.
